# *Helicobacter pylori *phagosome maturation in primary human macrophages

**DOI:** 10.1186/1757-4749-3-3

**Published:** 2011-03-23

**Authors:** Glenn N Borlace, Hilary F Jones, Stacey J Keep, Ross N Butler, Doug A Brooks

**Affiliations:** 1 Mechanisms in Cell Biology and Disease Research Group, Sansom Institute for Health Research, School of Pharmacy and Medical Sciences, University of South Australia, South Australia, Australia; 2 Department of Paediatrics and Reproductive Health, University of Adelaide, South Australia, Australia

## Abstract

**Background:**

*Helicobacter pylori *(*H. pylori*) is a micro-aerophilic, spiral-shaped, motile bacterium that is the principal cause of gastric and duodenal ulcers in humans and is a major risk factor for the development of gastric cancer. Despite provoking a strong innate and adaptive immune response in the host, *H. pylori *persists in the gastric mucosa, avoiding eradication by macrophages and other phagocytic cells, which are recruited to the site of infection. Here we have characterised the critical degradative process of phagosome maturation in primary human macrophages for five genotypically and phenotypically distinct clinical strains of *H. pylori*.

**Results:**

All of the *H. pylori *strains examined showed some disruption to the phagosome maturation process, when compared to control *E. coli*. The early endosome marker EEA1 and late endosome marker Rab7 were retained on *H. pylori *phagosomes, while the late endosome-lysosome markers CD63, LAMP-1 and LAMP-2 were acquired in an apparently normal manner. Acquisition of EEA1 by *H. pylori *phagosomes appeared to occur by two distinct, strain specific modes. *H. pylori *strains that were negative for the cancer associated virulence factor CagA were detected in phagosomes that recruited large amounts of EEA1 relative to Rab5, compared to CagA positive strains. There were also strain specific differences in the timing of Rab7 acquisition which correlated with differences in the rate of intracellular trafficking of phagosomes and the timing of megasome formation. Megasomes were observed for all of the *H. pylori *strains examined.

**Conclusions:**

*H. pylori *appeared to disrupt the normal process of phagosome maturation in primary human macrophages, appearing to block endosome fission. This resulted in the formation of a hybrid phagosome-endosome-lysosome compartment, which we propose has reduced degradative capacity. Reduced killing by phagocytes is consistent with the persistence of *H. pylori *in the host, and would contribute to the chronic stimulation of the inflammatory immune response, which underlies *H. pylori*-associated disease.

## Background

*Helicobacter pylori (H. pylori) *is one of the most successful human pathogens, estimated to have infected half of the world's population [1]. *H. pylori *colonises the human stomach, is the primary cause of gastric and duodenal ulcers [2], and is a major risk factor for the development of gastric cancer [3,4]. *H. pylori *strains have been traditionally classified into two types on the basis of virulence factors [5]. Virulent type 1 strains secrete an active form of the vacuolating cytotoxin VacA (s1/m1) and possess the pathogenicity island *cag*PAI, whereas avirulent type 2 strains secrete an inactive form of VacA (s2/m2) and do not possess the *cag*PAI [6-8].

The hallmark of *H. pylori *infection is chronic inflammation of the gastric mucosa. *H. pylori *stimulates gastric epithelial cells to release pro-inflammatory cytokines, which recruit large numbers of neutrophils and macrophages to the site of infection [9-11]. Macrophages have been demonstrated to effectively phagocytose *H. pylori*, however in most cases this immune response fails to eradicate the bacterium from the host, suggesting that *H. pylori *has developed mechanisms to avoid phagocytic killing [12-15].

Although the predominant phagocytic cell type in the *H. pylori*-infected gastric mucosa is neutrophilic, significant numbers of macrophages are also found at the site of infection. Macrophages are critical regulators of the *H. pylori *immune response, acting as effector cells of both innate and adaptive immunity. Macrophages first facilitate the innate immune response by phagocytosing bacteria and then initiate the adaptive immune response through antigen presentation and the secretion of cytokines that direct lymphocyte recruitment and differentiation. *H. pylori *has been shown to be efficiently internalised by macrophages, but it avoids phagocytic killing. *In vitro *studies have shown that live *H. pylori *can be recovered from within human monocytic cell lines and primary human macrophages, up to 48 hours after infection [16-18]. Although the mechanism that *H. pylori *employs to avoid phagocytic killing is not completely understood, some disruptions to the normal process of phagosome maturation have been noted in *H. pylori*-infected macrophages.

The normal process of phagosome maturation has been characterised as the step wise interaction of phagosomes with early endosomes, late endosomes and lysosomes [19,20]. Newly formed phagosomes interact with early endosomes, gaining early endosome markers such as Rab5 and EEA1. After 10-30 minutes, the early endosome markers are lost from the phagosome and are replaced by late endosome markers, such as Rab7 and CD63. After approximately one hour, the late endosome markers are lost and phagosomes fuse with lysosomes to become phago-lysosomes, with a full complement of lysosomal hydrolytic enzymes and maximum degradative capacity. Phago-lysosomes can best be characterised by the presence of the lysosome associated membrane proteins LAMP-1 and LAMP-2 [21].

It has been hypothesized that *H. pylori *arrests the normal phagosome maturation process at a stage prior to the phagosome reaching full degradative capacity, thus allowing for prolonged bacterial survival in a non-lethal intracellular niche [22]. Previous reports on the composition of the *H. pylori *compartment in macrophages have provided evidence for differences between phagosomes containing virulent type 1 and avirulent type 2 strains of *H. pylori *[16]. Avirulent type 2 strains of *H. pylori *have been reported to undergo normal phagosome maturation, resulting in a fully degradative phago-lysosome compartment [16]. Conversely, virulent type 1 strains of *H. pylori *have been reported to disrupt phagosome maturation, and generate abnormal compartments termed megasomes [16,17,23,24]. Megasomes are believed to arise due to the homotypic fusion of *H. pylori *phagosomes, resulting in a communal compartment with less degradative capacity. Megasomes containing virulent type 1 strains of *H. pylori *have been reported to retain the early phagosome marker coronin 1 and the early endosome marker EEA1, but exclude the lysosomal marker LAMP-1 [16,17,24]. The specific *H. pylori *virulence factors responsible for prolonged intracellular survival and disturbed phagosome maturation are beginning to be investigated. *In vitro *studies in mouse peritoneal macrophages and macrophage cell lines, utilising isogenic mutant strains of *H. pylori*, have demonstrated that VacA and urease were both essential for the prolonged survival of *H. pylori*, but only urease deletion mutants were unable to form megasomes [17,24].

To our knowledge, there has not been a systematic time-course study undertaken to characterise the *H. pylori *phagosome and this has led to potential misconceptions on *H. pylori *phagosome maturation. Utilising a set of markers to define early endosomes, late endosomes and lysosomes, we have characterised and compared the sequential steps in the phagosome maturation process for five genotypically and phenotypically distinct clinical isolates of *H. pylori*, and a control *E. coli *strain, using a primary human macrophage model of infection.

## Methods

### Bacterial strains and culture conditions

The *H. pylori *isolates used in this study were typed for the vacuolating cytotoxin (VacA) and *cag *pathogenicity island (*cag*PAI) virulence factors by polymerase chain reaction, and for the expression of CagA protein by Western analysis as previously described [18]. The SS1 (Sydney Strain 1) strain [25] had the *vacA *genotype s2/m2, and was positive for the *cag*PAI and expressed the CagA protein. NL101 was *vacA *s1a/m1, *cag*PAI positive, CagA negative. NL103 was *vacA *s2/m2, *cag*PAI negative, CagA negative. NL106 was *vacA *s2/m2, *cag*PAI positive, CagA negative. NL107 was *vacA *s1a/m1, *cag*PAI positive, CagA positive. The *E. coli *DH5α strain [26] was used as a control in this study.

Plate cultures of bacteria were grown on Columbia agar plates (Oxoid, Basingstoke, UK) containing 5% (v/v) horse blood (Institute of Medical and Veterinary Science (IMVS), Adelaide, South Australia). *E. coli *DH5α was incubated overnight at 37°C in aerobic conditions. *H. pylori *strains were routinely incubated for 3 days at 37°C under microaerophilic conditions (5% O_2_, 10% CO_2_). For the intracellular survival experiments, lysates from the *H. pylori*-infected macrophages were incubated for up to 5 days to allow sufficient time for visible colonies to be formed.

### Preparation of primary human macrophages

Human peripheral blood mononuclear cells (PBMCs) were isolated from whole blood by centrifugation on Ficoll-Paque™ PLUS (GE Healthcare, Sweden) gradients and cultured as previously described [18], but with the following adaptations for immunofluorescence experiments. PBMCs were plated onto 22 × 22 mm glass coverslips in 6 well tissue culture plates and incubated for 2 hours (37°C, 5% CO_2_) to allow for the monocytes to adhere. The non-adherent cells were then removed by aspiration and vigorous washing with phosphate buffered saline (PBS). The monocytes were cultured in Basal Eagle cell culture medium (MP Biomedicals, Irvine, CA) containing 20% (v/v) foetal bovine serum (JRH Biosciences, Lenexa, KS, USA), at 37°C in 5% CO_2 _for 7 days, to allow their differentiation into macrophages. Macrophage differentiation was routinely confirmed by immunofluorescence staining, using an antibody to the macrophage specific marker CD206 supplied by BD Biosciences Pharmingen, San Diego, CA, USA (data not shown).

### Bacterial infection of primary human macrophages

Primary human macrophages were infected with either *H. pylori *or *E. coli *suspensions in cell culture medium (see above) at a multiplicity of infection of 50 bacteria per cell. Wet mounts of the *H. pylori *suspensions were examined microscopically on the day of infection to determine that the bacteria were of the viable spiral form. The viability of *H. pylori *and *E. coli *in the inocula was confirmed by viable counts of the 0.5 McFarland suspensions of *H. pylori *and *E. coli *(CFU/mL between 1 and 2 × 10^8 ^CFU/mL, data not shown). Contact between the bacteria and the macrophages was facilitated by centrifugation of the tissue culture plates at 1,000 *g *for 3 minutes at 20°C. Phagocytosis was initiated by incubation at 37°C in 5% CO_2 _and allowed to proceed for either 0, 15 or a maximum of 30 minutes. Extracellular bacteria were then removed by thorough washing with PBS (5 times using 3 mL per wash). The final washes were plated and incubated as above (for 5 days) to ensure no residual live bacteria remained outside of the macrophages after these treatments. This optimised infection protocol resulted in macrophages containing approximately 10-15 phagosomes per cell. For the *H. pylori *killing experiments; infected macrophages were cultured for 2 hours, 8 hours or 24 hours following the initiation of phagocytosis before being lysed (0.1% saponin, 15 minutes at 37°C), and the viable bacteria enumerated by scoring colonies of plated serial dilutions of the macrophage lysates. An additional 30 minute time-point was included for the *E. coli *killing experiments to confirm that the number of *E. coli *internalised by macrophages was comparable to that observed for *H. pylori*. All infections were performed in triplicate, with viable counts performed in duplicate. For the immunofluorescence experiments, infected macrophages were cultured for 0 minutes, 15 minutes, 30 minutes, 2 hours or 4 hours following the initiation of phagocytosis, before being fixed in 50% methanol/acetone (v/v) for 10 minutes on ice. Fixed coverslips were stored overnight at 4°C in PBS before immunofluorescence staining.

### Preparation of LAMP-1 antibody

Sheep anti-human LAMP-1 antibody was a kind gift from Debbie Lang, Children Youth and Women's Health Service (CYWHS), North Adelaide, South Australia. Recombinant human LAMP-1 was expressed and purified as previously described [27], then conjugated to diphtheria toxoid for immunisation (Mimotopes, Melbourne, Victoria). Polyclonal antibody production in sheep was by a standard immunisation regimen (IMVS, Veterinary Services Division, Gilles Plains, South Australia) and followed the Australian code of practice for the care and use of animals for scientific purposes. Briefly, a sheep was primed by multiple subcutaneous injections of a total of 1 mg of protein in Freund's Complete Adjuvant. Three subsequent injections of 1 mg of protein in Freund's Incomplete Adjuvant were given at 3 week intervals. Two weeks after the final injection the sheep was exsanguinated and the serum collected. The polyclonal antibody was purified by affinity chromatography.

### Immunofluorescence analysis

Coverslips were blocked with 5% (w/v) bovine serum albumin (BSA; Sigma-Aldrich, St. Louis, MO, USA) in PBS for 1 hour at room temperature. Primary and secondary antibody solutions for immune detection were prepared in 5% BSA in PBS. *H. pylori *was detected with a 1/100 dilution of caprylic acid purified rabbit anti-*H. pylori *serum [18] and *E. coli *was detected with 130 μg/mL of rabbit anti-*E. coli *serum (Dako, Glostrup, Denmark). The antibodies to endosome-lysosome markers were: mouse anti-human Rab5 (BD Biosciences Pharmingen, San Diego, CA, USA; 5 μg/mL), goat anti-human EEA1 (Santa Cruz Biotechnology, Santa Cruz, CA, USA; 4 μg/mL), mouse anti-Rab7 (Sigma-Aldrich; 2.2 μg/mL), mouse anti-human CD63 (kind gift from Andrew Zannetino, IMVS, Adelaide, South Australia, [28]; 5 μg/mL), sheep anti-human LAMP-1 (2.26 μg/mL), and mouse anti-human LAMP-2 (kind gift from Caroline Dean, CYWHS, North Adelaide, South Australia, [29]; 3.37 μg/mL). After incubation for 2 hours at room temperature, unbound primary antibodies were removed by washing 3 times with PBS. Primary antibodies to the bacteria were detected with a 1/100 dilution of FITC-conjugated donkey anti-rabbit antibody (Chemicon International, Millipore, Temecula, CA, USA). Primary antibodies to the endosome-lysosome markers were detected with a 1/500 dilution of Cy3-conjugated respective secondary antibody: either donkey anti-mouse (Rockland Immunochemicals, Gilbertsville, PA, USA), donkey anti-goat or donkey anti-sheep (Chemicon). After incubation for 1 hour at room temperature, unbound secondary antibodies were removed by washing as described above. The coverslips were mounted with ProLong Gold^® ^antifade containing DAPI (Invitrogen Corp. Carlsbad, CA, USA). Slides were incubated for 48 hours at room temperature in the dark before examination and imaging by confocal microscopy.

### Confocal microscopy and co-localisation analysis

For fluorescence imaging, confocal microscopy was performed using a Leica SP5 Laser Scanning Spectral Confocal Microscope (Adelaide Microscopy, University of Adelaide, South Australia). Macrophages containing 10-15 phagosomes were screened from each of two independent experiments and representative images were taken for co-localisation analysis. Images were collected and analysed using the Leica Application Suite for Advanced Fluorescence software (Leica Microsystems, Wetzlar, Germany). For co-localisation analysis, a scatter-plot, which correlated the colour and intensity of each pixel with respect to the contribution from each of the red and green channels, was generated for each image. Background threshold was set at 20% and the overlap coefficient was calculated as a statistical measurement of co-localisation. The overlap coefficient averages used to generate the graphs were determined from approximately 100 phagosomes for each treatment in duplicate experiments. Fluorescence images were processed to create the micrograph figures using Adobe Photoshop software (Adobe Systems Inc., San Jose, CA, USA).

### Ethics

The collection of human blood samples for the study of *H. pylori *was approved by the University of South Australia Human Research Ethics Committee (Ethics Protocol P129/08) and the Australian Red Cross Blood Service (Agreement Number 08-06SA-05).

## Results

### Bacterial killing by primary human macrophages

**Figure 1:**
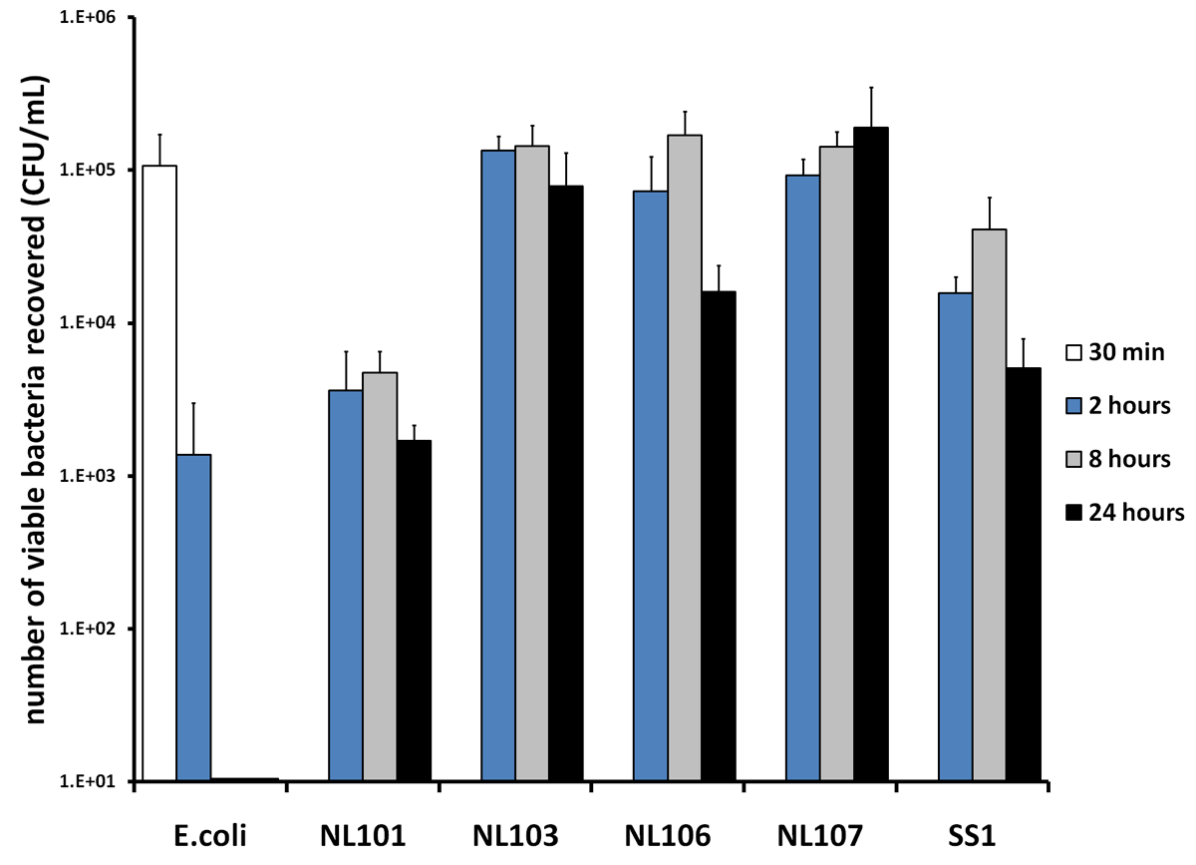
**Bacterial killing by primary human macrophages**. The ability of primary human macrophages to kill *E. coli *strain DH5α (*E. coli*) was compared to five *H. pylori *isolates (NL101, NL103, NL106, NL107 and SS1). The number of viable bacteria recovered from macrophages 30 minutes (open bars) two hours (blue bars), eight hours (grey bars) or 24 hours (closed bars) after infection is represented in colony forming units per millilitre (CFU/mL). Infected macrophage cultures were extensively washed to remove extra-cellular bacteria. Data is the mean of three independent experiments.

The numbers of viable bacteria recovered from primary human macrophages infected with either *E. coli *or *H. pylori *were determined over a 24 hour time course (Figure 1). At thirty minutes and two hours after infection, viable *E. coli *were recovered from infected primary human macrophages, but no viable bacteria were recovered eight and 24 hours after infection (Figure 1, *E. coli*). In contrast, for all of the *H. pylori *strains examined, viable bacteria were recovered from infected primary human macrophages at two, eight and 24 hours after infection (Figure 1, NL101, NL103, NL106, NL107 and SS1). This demonstrated that *E. coli *DH5α was efficiently killed and therefore a suitable control for phagosome maturation, in our primary human macrophage model of infection.

### Characterisation of phagosome maturation

**Figure 2:**
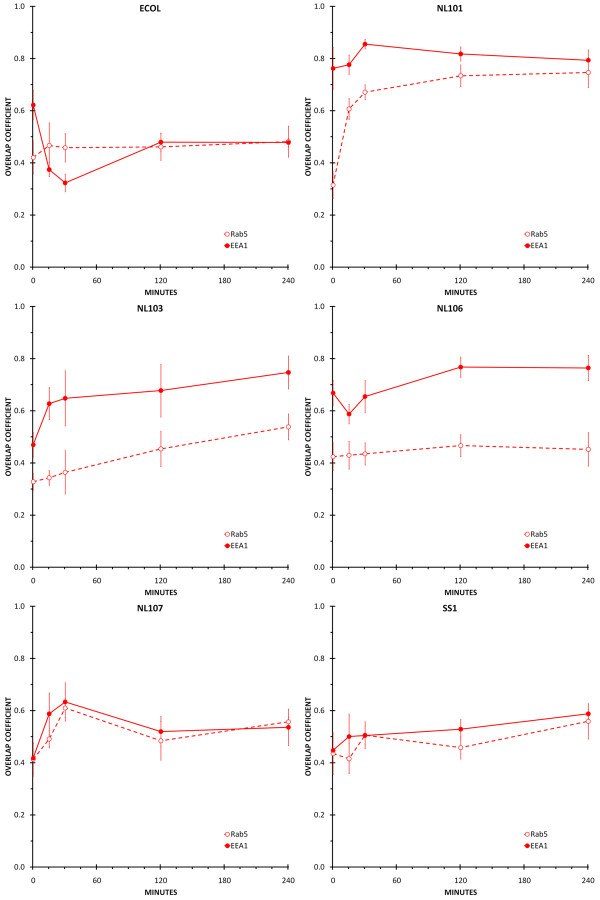
**Co-localisation of early endosome markers with *H. pylori *and *E. coli *phagosomes**. Individual panels show the amount of co-localisation between the early endosome markers Rab5 (open circle and dashed line) and EEA1 (closed circle and solid line) and *H. pylori *(NL101, NL103, NL106, NL107, SS1) or *E. coli *(ECOL) phagosomes. Time-points were 0 minutes, 15 minutes, 30 minutes, two hours and four hours following initiation of phagocytosis. Co-localisation between the bacteria (FITC) and early endosome markers (CY3) was quantified by calculating the overlap coefficients from confocal immunofluorescence images of macrophages containing approximately 100 phagosomes for each treatment, in duplicate.

**Figure 3:**
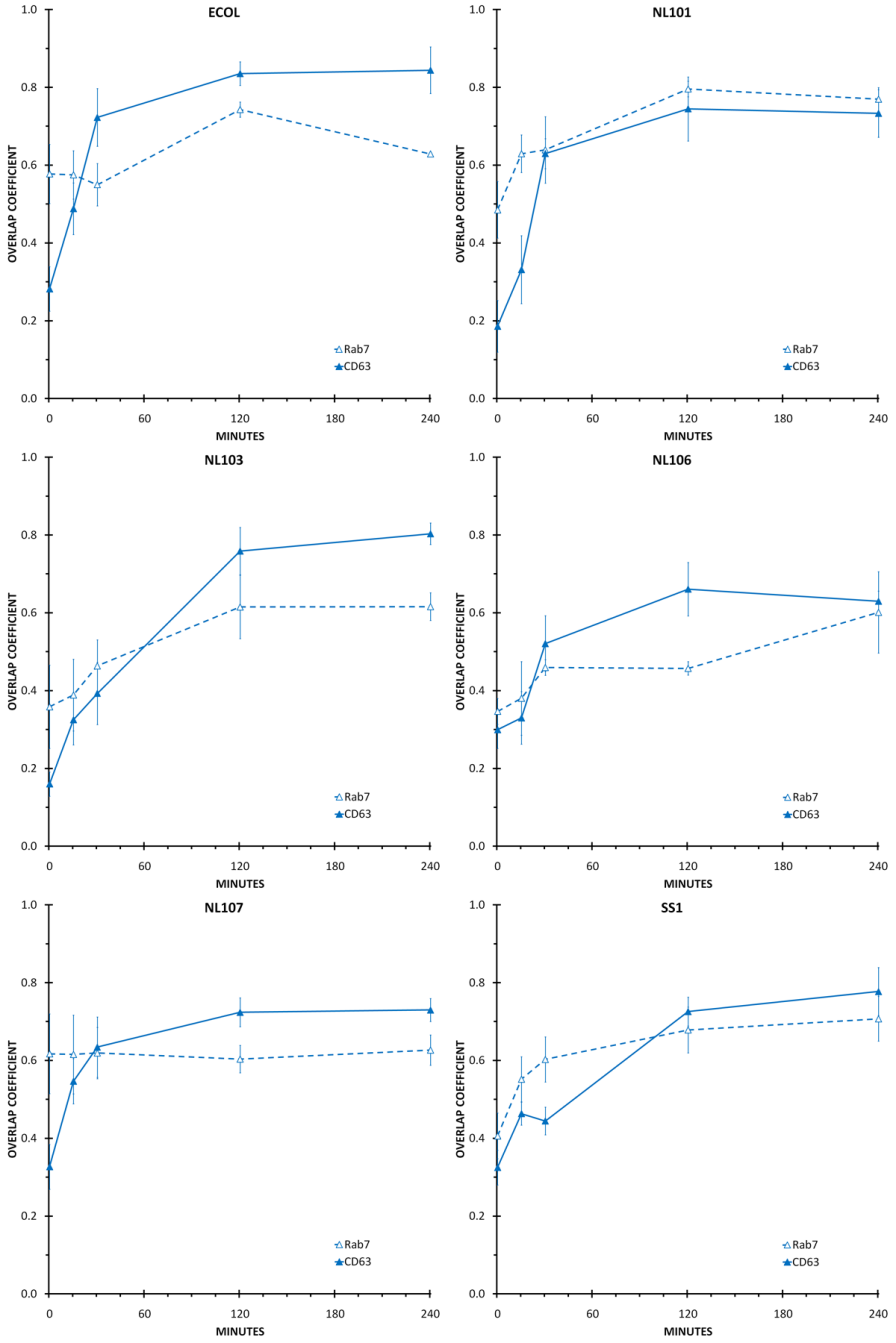
**Co-localisation of late endosome markers with *H. pylori *and *E. coli *phagosomes**. Individual panels show the amount of co-localisation between the late endosome markers Rab7 (open triangle and dashed line) and CD63 (closed triangle and solid line) and *H. pylori *(NL101, NL103, NL106, NL107, SS1) or *E. coli *(ECOL) phagosomes. Time-points were 0 minutes, 15 minutes, 30 minutes, two hours and four hours following initiation of phagocytosis. Co-localisation between the bacteria (FITC) and late endosome markers (CY3) was quantified by calculating the overlap coefficients from confocal immunofluorescence images of macrophages containing approximately 100 phagosomes for each treatment, in duplicate.

**Figure 4:**
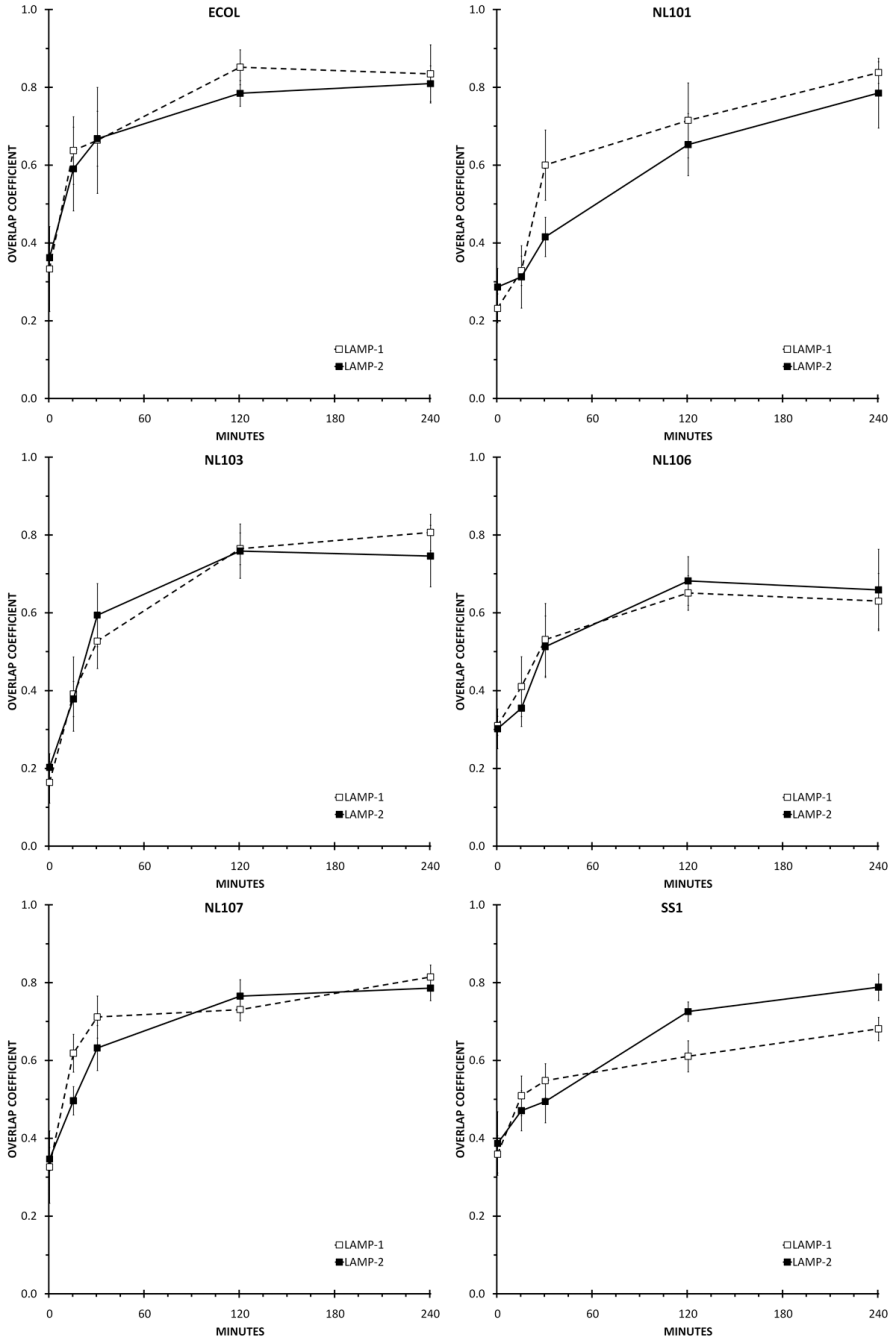
**Co-localisation of LAMP-1 andLAMP-2 with *H. pylori *and *E. coli *phagosomes**. Individual panels show the amount of co-localisation between the lysosome markers LAMP-1 (open square and dashed line) and LAMP-2 (closed square and solid line) and *H. pylori *(NL101, NL103, NL106, NL107, SS1) or *E. coli *(ECOL) phagosomes. Time-points were 0 minutes, 15 minutes, 30 minutes, two hours and four hours following initiation of phagocytosis. Co-localisation between the bacteria (FITC) and LAMP-1 and LAMP-2 markers (CY3) was quantified by calculating the overlap coefficients from confocal immunofluorescence images of macrophages containing approximately 100 phagosomes for each treatment, in duplicate.

The process of phagosome maturation in primary human macrophages was defined by determining the amount of Rab5, EEA1, Rab7, CD63, LAMP-1 or LAMP-2 that co-localised with *E. coli *DH5α or *H. pylori *NL101, NL103, NL106, NL107 and SS1 phagosomes at 0 minutes, 15 minutes, 30 minutes, two hours and four hours after the initiation of phagocytosis (Figures 2, 3, 4).

### Co-localisation of early endosome markers with *E. coli *phagosomes

Rab5 showed a consistently low amount of co-localisation with *E. coli *DH5α phagosomes, throughout the four hour time-course (Figure 2, ECOL). In contrast, there was a decrease in the amount of EEA1 that was co-localised with *E. coli *DH5α phagosomes from a high immediately after infection to a low at the 15 and 30 minute time-points. At two and four hours after infection, the amount of EEA1 co-localised with *E. coli *DH5α phagosomes was equivalent to that seen for Rab5 (Figure 2, ECOL).

### Co-localisation of early endosome markers with *H. pylori *phagosomes

There were strain specific differences observed in the acquisition of the early endosome markers by *H. pylori *phagosomes. *H. pylori *NL101 phagosomes showed a substantially higher amount of co-localisation with EEA1 than with Rab5 immediately after infection. This high level of EEA1 co-localisation was maintained throughout the four hour time course. The amount of Rab5 associated with *H. pylori *NL101 phagosomes increased at 15 and 30 minutes after infection before reaching a level similar to that seen for EEA1 at two hours and four hours after infection (Figure 2, NL101). *H. pylori *NL103 and NL106 phagosomes showed substantially higher co-localisation with EEA1 than with Rab5 throughout the four hour time-course (Figure 2, NL103 and NL106). In contrast, for *H. pylori *NL107 and SS1 phagosomes, there were similar levels of co-localisation with EEA1 and Rab5 throughout the four hour time-course. For *H. pylori *NL103 and NL106, Rab5 showed low level co-localisation with phagosomes throughout the four hour time-course (Figure 2, NL103) even though NL103 showed increasing levels of co-localisation with Rab5 throughout the time course (Figure 2, NL103). For *H. pylori *NL107, Rab5 and EEA1 co-localisation with phagosomes increased over the first thirty minutes following infection, decreased between 30 minutes and two hours, before stabilising at a low level at two to four hours after infection (Figure 2, NL107). For SS1, there were only low levels of co-localisation with Rab5 and EEA1 throughout the time course and although the amount of co-localisation increased at each time point, the maximum co-localisation at four hours was only low (Figure 2, SS1).

### Co-localisation of late endosome markers with *E. coli *phagosomes

Immediately after infection, *E. coli *DH5α phagosomes showed some co-localisation with Rab7. The amount of Rab7 associated with *E. coli *DH5α phagosomes remained steady at 15 and 30 minutes after infection, before increasing to a maximum at the two hour time-point and decreasing at four hours after infection (Figure 3, ECOL). There was little or no association of CD63 with *E. coli *DH5α phagosomes at time zero after infection. The amount of CD63 that was co-localised with *E. coli *DH5α phagosomes increased at the 15 minute, 30 minute and two hour time-points, to an amount that was maintained at four hours after infection (Figure 3, ECOL).

### Co-localisation of late endosome markers with *H. pylori *phagosomes

There were also strain specific differences in late endosome marker acquisition by *H. pylori *phagosomes. *H. pylori *NL107 phagosomes showed their maximum amount of co-localisation with Rab7 immediately after infection and this was maintained throughout the four hour time course (Figure 3, NL107). In contrast, there was little or no co-localisation of Rab7 with *H. pylori *NL101, NL103, NL106 and SS1 phagosomes immediately after infection. For NL101 and SS1 the amount of Rab7 associated with phagosomes increased at 15 minutes after infection and reached a maximum at 2 hours after infection and maintained this level of association at the four hour time point (Figure 3, NL101 and SS1). For NL103, it appeared that Rab7 did not become associated with phagosomes until the two hour time point, but this maximum amount of co-localisation was then maintained at the four hour time point (Figure 3, NL103). For NL106, there was an increase in the amount of Rab7 associated with phagosomes at 30 minutes, however this was at lower levels than for other strains and maximum co-localisation was not achieved until four hours after infection (Figure 3, NL106). All of the *H. pylori *strains examined showed a similar pattern for the phagosomal acquisition of CD63. There was little or no co-localisation of CD63 with phagosomes immediately after infection. The amount of CD63 co-localised with phagosomes increased at either 30 minutes (*H. pylori *strains NL101, NL106 and NL107), or two hours after infection (NL103 and SS1) and these maxima were maintained until the four-hour time point (Figure 3).

### Co-localisation of LAMP-1 and LAMP-2 with *E. coli *phagosomes

Over the four hour time-course, LAMP-1 and LAMP-2 showed a similar pattern of co-localisation with *E. coli *DH5α phagosomes as that observed for CD63. The amount of LAMP-1 and LAMP-2 associated with *E. coli *phagosomes increased at each time-point over the first two hours, from a low immediately after infection to a high that was maintained between two and four hours after infection (Figure 4, ECOL).

### Co-localisation of LAMP-1 and LAMP-2 with *H. pylori *phagosomes

The pattern of lysosome marker acquisition by *H. pylori *phagosomes was similar to that observed for CD63 for all of the *H. pylori *strains examined. At time zero after infection, LAMP-1 and LAMP-2 showed little or no co-localisation with phagosomes. The amount of LAMP-1 and LAMP-2 co-localised with phagosomes steadily increased at the 15 minute, 30 minute and two hour time-points post-infection, and reached a maximum between two and four hours after infection (Figure 4).

### *H. pylori *internalised into macrophages were trafficked to the peri-nuclear region at different times for each strain

**Figure 5:**
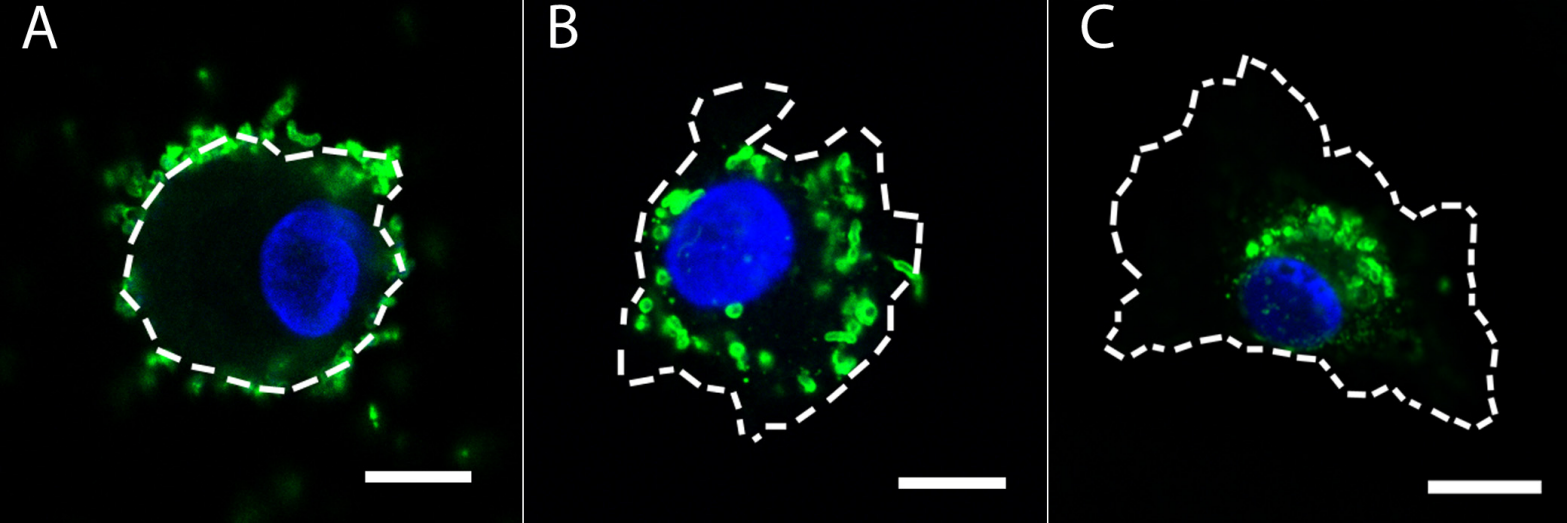
**Intracellular location of *H. pylori *in infected macrophages**. Confocal micrographs of primary human macrophages infected with *H. pylori *NL103 immune stained with rabbit anti-*H. pylori *serum/donkey anti-rabbit FITC conjugate (green) and nuclear DNA stained with DAPI (blue). The size and shape of macrophages are defined by dashed white lines. *H. pylori *are located at the cell surface/periphery at 0 and 15 minutes after infection (A), at an intermediate position between the cell periphery and the nucleus (cytoplasmic) 30 minutes after infection (B), and in a peri-nuclear location two and four hours after infection (C). Scale bar = 5 μm.

Primary human macrophages were infected with *H. pylori *and changes in the intracellular location of the bacteria were tracked over a four hour time-course using confocal immunofluorescence microscopy. During the course of infection bacteria were observed to have been internalised and trafficked from the cell surface/periphery, through the cytoplasm to a destination at the peri-nuclear region (Figure 5). The rate of trafficking from the cell surface to the peri-nuclear region varied for the different *H. pylori *strains (Table 1). *H. pylori *NL103 and NL106 appeared to have the slowest transit times for the *H. pylori *strains examined, not reaching the peri-nuclear region until the two-hour time-point. *H. pylori *NL101 and SS1 phagosomes were detected in the peri-nuclear region by 30 minutes after infection and the fastest transit time was observed for *H. pylori *NL107, which had been re-located to the peri-nuclear region by 15 minutes after infection.

**Table 1:** Relative position of intracellular bacteria in macrophages over a four hour time course

		Bacterial Strain					
		NL101	NL103	NL106	NL107	SS1	*E. coli*
**time after infection**	**0 min**	CS/P	CS/P	CS/P	CS/P	CS/P	CS/P
	**15 min**	Cy	CS/P	CS/P	PN	Cy	Cy
	**30 min**	PN	Cy	Cy	PN	PN	Cy
	**2 hr**	PN	PN	PN	PN	PN	PN
	**4 hr**	PN	PN	PN	PN	PN	PN

Intracellular position of *H. pylori *strains NL101, NL103, NL106, NL107, SS1 and *E. coli *DH5α in primary human macrophages 0 minutes, 15 minutes, 30 minutes, 2 hours and 4 hours after infection. CS/P: cell surface/periphery, Cy: cytoplasmic, PN: peri-nuclear.

### *H. pylori *promotes formation of megasomes

**Figure 6:**
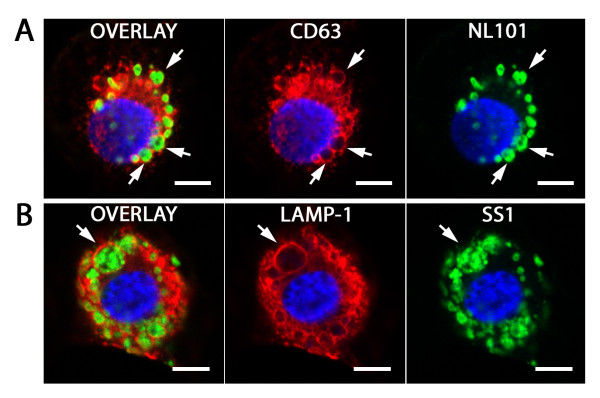
***H. pylori *megasomes**. Confocal immunofluorescence micrographs of megasomes in primary human macrophages 30 minutes after infection with *H. pylori *strain NL101 (A) or NL107 (B). Infected macrophage are immune stained with rabbit anti-*H. pylori *serum/donkey anti-rabbit IgG FITC conjugate (green), mouse anti-CD63/donkey anti-mouse IgG CY3 conjugate or sheep anti-LAMP-1/donkey anti-sheep IgG CY3 conjugate (red) and nuclear DNA stained with DAPI (blue). Arrows designate megasomes containing multiple intact *H. pylori *in overlay and single channels. Scale bar = 5 μm.

A distinctive feature of *H. pylori *infected macrophages was the appearance of megasomes, containing multiple intact *H. pylori *(Figure 6). For each of the *H. pylori *strains examined, the megasomes had a similar marker composition to that observed for the phagosomes containing a single bacterium. Thus, megasomes were characterised by the retention of endosome markers EEA1, Rab7 and CD63, despite recruiting the lysosome associated membrane proteins LAMP-1 and LAMP-2 in an apparently normal manner. However, in different bacterial strains megasomes appeared at different time-points (Table 2). They were detected at 15 minutes after infection for *H. pylori *strain NL107, at 30 minutes for *H. pylori *strains NL101 and SS1 and not until two hours for *H. pylori *strains NL103 and NL106.

**Table 2:** The appearance of megasomes in *H. pylori*-infected primary human macrophages correlates with acquisition of EEA1 and Rab7 and the intracellular position of the bacteria.

*H. pylori *strain	Time after infection	EEA1 colocalisation	Rab7 colocalisation	Intracellular position	Megasomes
**NL101**	**0 min**	+	-	CS/P	-

	**15 min**	+	+	Cy	-

	**30 min**	+	+	PN	+

	**2 hr**	+	+	PN	+

	**4 hr**	+	+	PN	+

**NL103**	**0 min**	+	-	CS/P	-

	**15 min**	+	-	CS/P	-

	**30 min**	+	-	Cy	-

	**2 hr**	+	+	PN	+

	**4 hr**	+	+	PN	+

**NL106**	**0 min**	+	-	CS/P	-

	**15 min**	+	-	CS/P	-

	**30 min**	+	-	Cy	-

	**2 hr**	+	-	PN	+

	**4 hr**	+	+	PN	+

**NL107**	**0 min**	-	+	CS/P	-

	**15 min**	+	+	PN	+

	**30 min**	+	+	PN	+

	**2 hr**	+	+	PN	+

	**4 hr**	+	+	PN	+

**SS1**	**0 min**	-	-	CS/P	-

	**15 min**	+	+	Cy	-

	**30 min**	+	+	PN	+

	**2 hr**	+	+	PN	+

	**4 hr**	+	+	PN	+

*H. pylori *clinical strains: NL101, NL103, NL106, NL107, SS1. Time after infection of primary human macrophages: 0 minutes, 15 minutes, 30 minutes, 2 hours and 4 hours. Intracellular position of *H. pylori *strains in infected primary human macrophages: CS/P; cell surface/periphery, Cy: cytoplasmic, PN: peri-nuclear. Association of EEA1/Rab7 with *H. pylori *phagosomes and appearance of megasomes is either present (+) or absent (-).

## Discussion

*H. pylori *infection is characterised by chronic inflammation of the gastric mucosa, which results in the recruitment of large numbers of macrophages and other phagocytic cells. However, the persistence of *H. pylori *in the host indicates that this immune response fails to clear the infection. *In vitro *studies have demonstrated that macrophages are unable to eradicate phagocytosed *H. pylori*, and it has been postulated that this could be due to impaired phagosome maturation [16-18,24,30]. Here we present a systematic time-course study of the acquisition of early endosome, late endosome and lysosome markers to accurately define the sequence of the phagosome maturation process in five clinical strains of *H. pylori*.

In the first stage of the phagosome maturation process, phagosomes normally recruit markers characteristic of early endosomes. Here we report that the early endosome marker EEA1 was recruited to *H. pylori *phagosomes in two distinct modes, either acquiring similar amounts of Rab5 and EEA1 or larger amounts of EEA1 relative to Rab5. This suggested that some strains of *H. pylori *could acquire EEA1 independently of Rab5. The differences observed in the recruitment of Rab5 and EEA1 to *H. pylori *phagosomes correlated with the CagA typing of the bacterial strains. The *H. pylori *strains that were negative for CagA (NL101, NL103 and NL106) appeared to recruit EEA1 independently of Rab5, whereas the CagA positive *H. pylori *strain NL107 appeared to simultaneously recruit and lose Rab5 and EEA1 over the time course. The *H. pylori *SS1 strain produces a CagA protein that does not induce IL-8 production from epithelial cells [31] and recruited only small amounts of Rab5 and EEA1 over the time course. For the CagA negative strains, there was a high level of EEA1 co-localisation over the time course, despite the phagosomes also gaining the late endosome markers Rab7 and CD63. In comparison, the CagA positive strains either gained or lost their association with EEA1 (NL107) or gained only very low levels of EEA1 over the four hour time course (SS1). This differential recruitment of either early endosomes or early endosome trafficking machinery to *H. pylori *phagosomes could modulate the normal functioning of the endocytic compartments involved in for example, antigen presentation and cytokine secretion, two functions of endosome-lysosome compartments that have been implicated in the immune response to *H. pylori *infection. While the mechanism connecting CagA and EEA1 acquisition is unclear, there is an epidemiological association between bacterial strains that produce CagA and the more severe *H. pylori *disease outcomes, such as gastric cancer [32].

The next stage in the phagosome maturation process involves the loss of early endosome markers from the phagosome and concomitant gain of late endosome markers [20]. Despite the strain specific differences that were observed in the gain and loss of EEA1, all of the *H. pylori *strains examined showed abnormal retention of the late endosome marker Rab7 on their phagosomes, when compared to *E. coli*. In addition, there were strain specific differences observed in the timing of Rab7 recruitment to *H. pylori *phagosomes. Rab7 was acquired by *H. pylori *NL107 phagosomes immediately after infection, by *H. pylori *NL101 and SS1 phagosomes 15 minutes after infection, by *H. pylori *NL106 phagosomes 30 minutes after infection and by *H. pylori *NL103 phagosomes two hours after infection. These differences in the rate of Rab7 acquisition directly matched the transit times of phagosomes to the peri-nuclear region. This was consistent with the role of Rab7 to recruit downstream effectors that enable endosomes and lysosomes to attach to the dynein/dynactin motor complex, for trafficking along microtubules [33,34]. For all of the *H. pylori *strains examined, both Rab7 and CD63 were retained by phagosomes throughout the time course. This was not the case for *E. coli*, which showed a reduction in the amount of phagosome associated Rab7 after the two hour time-point. The retention of early and/or late endosome markers by *H. pylori *phagosomes represented a significant departure from the normal phagosome maturation process.

The final stage in the phagosome maturation process is normally characterised by the loss of late endosome markers and fusion with lysosomes [20]. Previous studies have suggested that *H. pylori *phagosomes acquire LAMP-1 differently in various cell model systems. In the transformed macrophage-like cell lines RAW264.7, J774 and THP-1, *H. pylori *phagosomes have been reported to be LAMP-1 negative, whereas in primary mouse peritoneal macrophages, wild type *H. pylori *are reported to acquire limited amounts of LAMP-1 [17,24]. Here, using primary human macrophages, we observed that *H. pylori *phagosomes acquired the lysosome associated membrane proteins LAMP-1 and LAMP-2 with the same kinetics as observed for *E. coli *phagosomes, despite retaining high levels of early and late endosome markers. Taken together these results indicated that while there appeared to be normal recruitment of early endosomes and late endosomes-lysosomes by *H. pylori *phagosomes, there appeared to be aberrant recovery of early and late endosomes during the phagosome maturation process. This blockage in endosome fission resulted in the formation of hybrid phagosome-endosome-lysosome compartments. While it is possible to conclude that there could be two distinct sub-populations of phagosomes positive for either early or late endosome markers, the high amounts of co-localisation suggest that this was unlikely. The retention of early and late endosomes by *H. pylori *phagosomes, coupled with normal phagosome-lysosome fusion, could result in larger phagosome compartments. These hybrid compartments would not be expected to have the same degradative properties as normal phagosomes, due to the increased size and reduced concentration of lysosomal hydrolases. This reduced phagosome degradative capacity could be responsible for the failure of macrophages to eradicate *H. pylori*.

Megasomes containing multiple intact bacteria were observed for all of the *H. pylori *strains examined in this study, and showed the same marker composition as phagosomes containing a single bacterium, in particular, the retention of EEA1. The timing differences observed in megasome formation for different *H. pylori *strains also correlated with the timing of Rab7 acquisition by phagosomes and the traffic of phagosomes to the peri-nuclear region (Table 2). This confirmed and extended the model for megasome formation proposed by Schwartz and Allen [24]. EEA1/Rab7 positive *H. pylori *phagosomes would be transported along the microtubule network to the peri-nuclear region of the cell, in a Rab7 dependent manner. The juxtaposition of EEA1 positive phagosomes in the peri-nuclear region would then facilitate tethering and phagosome fusion [35], in a manner analogous to the homotypic fusion of early endosomes [36]. While the retention of EEA1 by megasomes has been reported previously, the acquisition of LAMP-1 by megasomes was not described in a previous study [17]. Moreover, all strains of *H. pylori *were observed to generate megasomes in the current study, in contrast to previous reports of only type 1 strains of *H. pylori *forming megasomes [16,17]. These discrepancies may be explained by different rates of early and late endosome marker acquisition by each *H. pylori *strain, and consequently the timing of megasome formation. The *H. pylori *in phagosomes and megasomes appeared to have a rounded appearance and *H. pylori *is known to take on a viable, but non-culturable coccoid form in unfavourable environments [37]. However, despite the coccoid appearance, viable *H. pylori *were cultured from the macrophages after 2, 8 and 24 hours following infection.

There are several different strategies employed by intracellular pathogens to alter phagosome maturation and avoid phagocytic killing. *Mycobacterium tuberculosis *(*M. tuberculosis*) arrests phagosome maturation at an early stage - it recruits Rab5, but blocks the recruitment of Rab5 effectors [38,39]. *Legionella pneumophila *establishes a replicative niche within an endoplasmic-reticulum derived compartment [40] and *Coxiella burnetii *(the causative agent of Q fever) alters its intracellular compartment to resemble an autophagosome [41,42]. In contrast, *H. pylori *generates a unique, hybrid phagosome-endosome-lysosome compartment that retains some degradative capacity. This degradative capacity would account for the strong humoral immune response that *H. pylori *evokes in the host [43], as antigen presentation is critically reliant upon degradation of phagosomal cargo. The limited degradative capacity of phagosomes would be consistent with the long term persistence of *H. pylori *in the host and the survival of residual numbers of *H. pylori *in infected macrophages [16-18,23,24,30].

## Conclusions

The normal phagosome maturation process was disrupted in primary human macrophages that had been infected by *H. pylori*. While there appeared to be normal recruitment of early endosomes, late endosomes and lysosomes by *H. pylori *phagosomes, there was aberrant recovery of endosomes during the phagosome maturation process. These disruptions of phagosome maturation could contribute to the persistence of *H. pylori *in the host and chronic stimulation of the inflammatory immune response; a pre-cursor to all *H. pylori *associated diseases. Identifying the molecular mechanism that *H. pylori *uses to avoid macrophage-mediated killing could realise a strategy to reinstate the ability of the immune system to clear this pathogenic bacterium.

## Competing interests

The authors declare that they have no competing interests.

## Authors' contributions

GNB, HFJ and DAB conceived of the study. GNB, HFJ, SJK, RNB and DAB participated in design of the study and interpretation of the results. GNB carried out the *in vitro *infections, bacterial killing assays, immunofluorescence experiments, confocal microscopy and co-localisation analysis. GNB and DAB drafted the manuscript. All authors read and approved the final manuscript.
